# The Wired Patient: Patterns of Electronic Patient Portal Use Among Patients With Cardiac Disease or Diabetes

**DOI:** 10.2196/jmir.3157

**Published:** 2015-02-20

**Authors:** James Brian Jones, Jonathan P Weiner, Nirav R Shah, Walter F Stewart

**Affiliations:** ^1^Research, Development and DisseminationSutter HealthWalnut Creek, CAUnited States; ^2^Center for Population Health Information TechnologyDepartment of Health Policy and ManagementJohns Hopkins Bloomberg School of Public HealthBaltimore, MDUnited States; ^3^Kaiser Permanente Southern CaliforniaPasadena, CAUnited States

**Keywords:** electronic health record, eHealth, patient Web portal, electronic patient portal, personal health record

## Abstract

**Background:**

As providers develop an electronic health record–based infrastructure, patients are increasingly using Web portals to access their health information and participate electronically in the health care process. Little is known about how such portals are actually used.

**Objective:**

In this paper, our goal was to describe the types and patterns of portal users in an integrated delivery system.

**Methods:**

We analyzed 12 months of data from Web server log files on 2282 patients using a Web-based portal to their electronic health record (EHR). We obtained data for patients with cardiovascular disease and/or diabetes who had a Geisinger Clinic primary care provider and were registered “MyGeisinger” Web portal users. Hierarchical cluster analysis was applied to longitudinal data to profile users based on their frequency, intensity, and consistency of use. User types were characterized by basic demographic data from the EHR.

**Results:**

We identified eight distinct portal user groups. The two largest groups (41.98%, 948/2258 and 24.84%, 561/2258) logged into the portal infrequently but had markedly different levels of engagement with their medical record. Other distinct groups were characterized by tracking biometric measures (10.54%, 238/2258), sending electronic messages to their provider (9.25%, 209/2258), preparing for an office visit (5.98%, 135/2258), and tracking laboratory results (4.16%, 94/2258).

**Conclusions:**

There are naturally occurring groups of EHR Web portal users within a population of adult primary care patients with chronic conditions. More than half of the patient cohort exhibited distinct patterns of portal use linked to key features. These patterns of portal access and interaction provide insight into opportunities for electronic patient engagement strategies.

## Introduction

The adoption of health information technology (HIT), particularly electronic health records (EHR) and personal health records (PHR), is widely viewed as a critical step towards achieving improvements in the quality and efficiency of the US health care system. The rapid growth of the Internet has made it possible for patients to independently obtain medical information and increasingly obtain health care on a temporally asynchronous basis. The Internet is widely used for seeking health-related information and patients are demanding access to physician email, Web-based appointment scheduling, and laboratory results online [1-4]. In response, structured health systems and academic centers with EHR-based HIT infrastructures are implementing Web-based patient portals that give patients access to their EHRs and other electronic care functions [5,6]. There is an expectation that these new approaches to clinical interaction increase access and reduce costs. Relatively little is known about how patients electronically access their provider’s HIT system via the portal. Deploying and maintaining a portal requires substantial investments of time, capital, and technical resources. Understanding how users interact with the portal is fundamentally important to evolving features that meet user needs and incorporate electronically supported services into existing clinician and patient workflows. Indeed, current and proposed criteria for “meaningful use” include functionality currently available in many portals. As these criteria are finalized, they should be informed by experience with the first generation of portals now in use [7,8]. Moreover, Web portal experience will have considerable implications for patient controlled personal health records (PHRs) as they are integrated with provider-based EHR systems.

What is currently known about portal users, or more broadly, individuals who use the Internet for health and health care-related purposes, is based mainly on self-reported patient attitudes and expectations [9-13], with few empirical assessments of actual use [14-19]. A recent review found little evidence to support the association between portal use and improvement in patient care. The authors found that few studies actually provided usage information, and the degree to which patients “exploited the offered functionalities” is unknown [20]. Relatively little is known about actual use because most portal interactions are difficult to track longitudinally at the individual level. To address this gap in our understanding of portal use, we used the audit trail function of the Web server transaction log file data from the Geisinger Clinic’s portal to understand how patients actually used the system over a long-term (12-month) period. Similar analyses have been used to improve the utility of other types of information systems such as medical library websites [21-25]. We hypothesized that patients have different motivations and expectations for use that are manifest in their unique transaction patterns.

## Methods

### Overview

This study is a secondary analysis of administrative and EHR data for a cohort of 4945 Geisinger Clinic (GC) patients with cardiovascular disease and/or diabetes. GC is a network of more than 40 community practice sites in Central and Northeastern Pennsylvania, each of which uses the EpicCare EHR. The analysis cohort consisted of 3297 patients who were users of “MyGeisinger”, a Web-based electronic patient portal, and a comparison-matched group of 1648 patients who did not use MyGeisinger. This research was approved by the Institutional Review Boards of both Geisinger Health System and the Johns Hopkins University, and patient anonymity was strictly maintained.

### MyGeisinger Patient Portal

**Figure 1:**
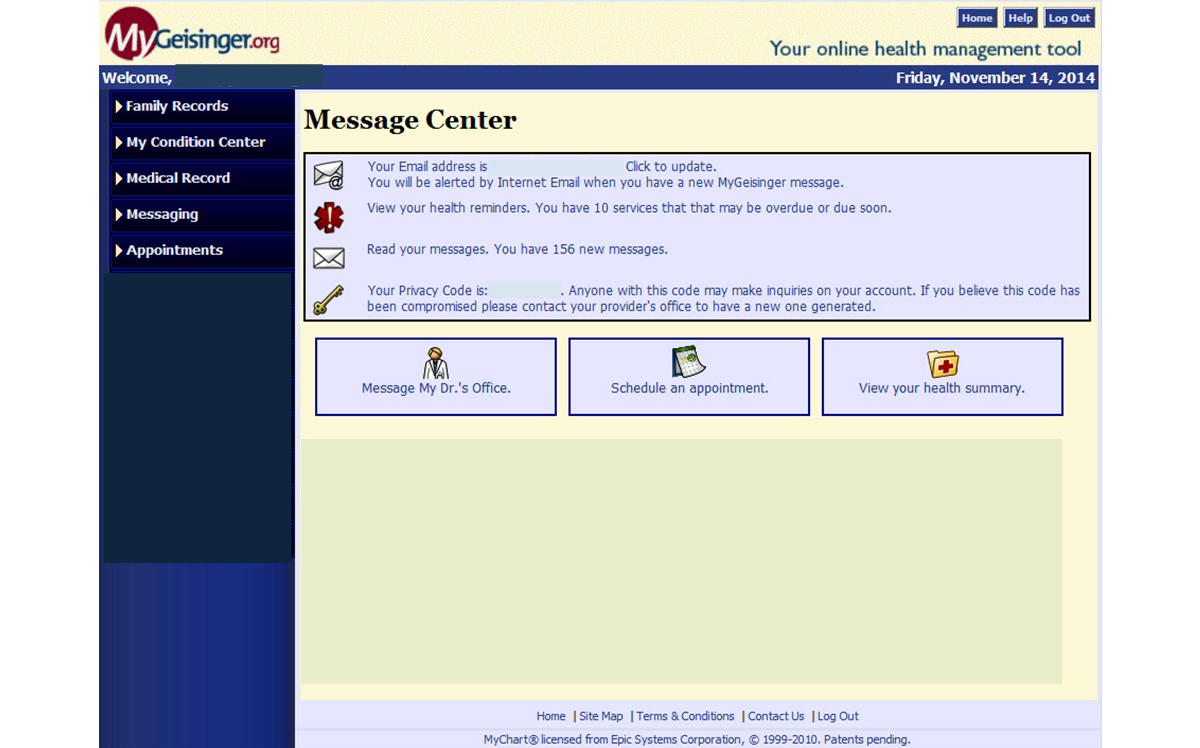
Screenshot of the MyGeisinger Patient Portal.

MyGeisinger is a secure, no-cost (to the patient) Web-based portal that allows a patient to access portions of their EHR (Figure 1). MyGeisinger can be used to access medical record information including medications, allergy, and problem lists; view preventive health reminders, provider information, and details of previous office visits; review, track, and graph laboratory test results and clinical measures (eg, blood pressure, weight); and interact with a provider via secure messaging. Patients can also use MyGeisinger to complete administrative tasks (eg, refilling medications, scheduling appointments, requesting referrals). MyGeisinger use is voluntary. The availability of these functions was consistent over the study period. Information is available in all clinic sites. To register, patients can either register at a kiosk in a GC site or request an account online, after which a letter with an activation code and instructions for completing the registration process online is mailed to their home address.

### Study Population

The analysis cohort consisted of patients who met the following inclusion criteria: (1) had a confirmed diagnosis, by International Classification of Diseases (ICD)-9 codes of diabetes, heart failure, and/or cardiovascular disease, (2) had an assigned primary care physician (PCP) in a GC community practice site, (3) had a visit with their PCP in the prior year, and (4) were registered users of the MyGeisinger portal. For comparison purposes, we also identified a matched (based on age, sex, and comorbid conditions) random sample of patients who met the first three inclusion criteria but had not registered to use MyGeisinger.

### Data Sources

The two sources of data used in this study were MyGeisinger Web server log files and Geisinger’s electronic health record.

All patient level MyGeisinger usage and interactions (ie, accessing a specific function by clicking on a link within MyGeisinger) are automatically recorded and time stamped in the log files maintained by the MyGeisinger Web server. For this study, we used MyGeisinger server logs from November 1, 2005, through October 31, 2006.

Information obtained from the EHR included body mass index (BMI), age, sex, comorbidities, and laboratory values relevant to chronic disease care (eg, HbA1c, low and high density lipoprotein values, blood pressure).

### Analysis

We approached the analysis in four steps. First, we used Web server log files to obtain detailed portal use information on a cohort of MyGeisinger users. Second, we developed a set of variables that quantitatively described the frequency, intensity, and types of portal use. Third, in order to determine whether there were similar groups of portal users, we used factor analysis to reduce the number of variables and then performed a cluster analysis to identify similar types of portal users. Fourth, to characterize the resulting clusters, we used a separate data source that included demographic and limited data from the EHR to profile the clusters.

### MyGeisinger Log Files

For each patient, the log file was transformed into a longitudinal series of records for the 12-month study period, where each record corresponds to a discrete portal session. A portal “session” begins when a patient logs in with a username and password and ends when the patient logs out or is inactive for more than 20 minutes (a “time-out”). Study participants for whom longitudinal data were unavailable (ie, ≤1 session during the study period) were considered “non-users” and excluded from the analysis. Multiple sessions were allowed per day or “hit-day” [26] (ie, a day with at least one portal session). In some cases, sessions recorded in the log file occurred in very close proximity to one another (ie, logout followed by login after a very short duration). For analytic purposes, we assumed that sessions in very close temporal proximity (≤3 minutes apart) were indicative of a single instance of portal activity and combined them accordingly.

**Figure 2:**
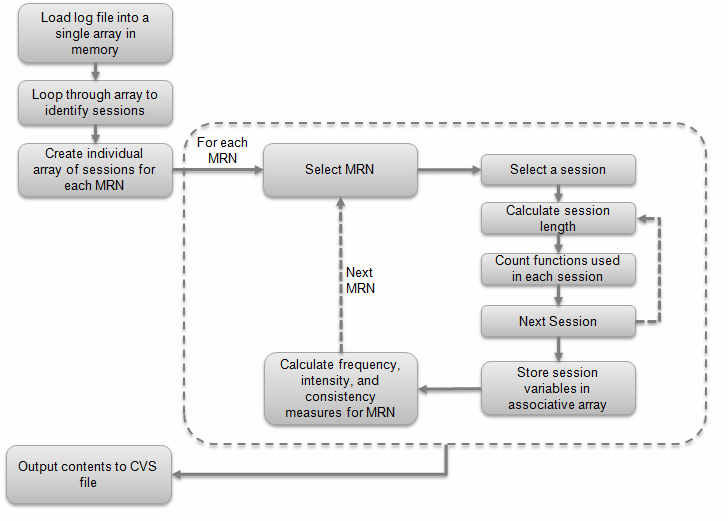
Summary of the process for parsing the log file using Perl. MRN: medical record number.

For each session, variables were created to quantify the length of the session (with adjustments made to account for time-outs) and to count the number of times each function (eg, checking lab results, emailing a physician) was used over the course of the study period. In this context, “use” of a function meant that a patient clicked on a link on the main MyGeisinger menu (eg, “Lab Results”) or a link available from within a specific menu option (eg, a link to review a specific lab result). Patients were able to access each function multiple times during a session. For each patient, we counted each time a link was clicked and summed these at the session level for each function. In addition, we created variables to describe the frequency, consistency, intensity, and duration of portal use. Portal transactions were classified as administrative (ie, appointment-related functions, driving directions to a Geisinger Clinic, provider details, proxy functions, and referral functions) or otherwise categorized as clinical. We counted the total number of administrative and clinical transactions across all sessions in the study period and calculated the administrative-to-care ratio (a ratio >1.0 indicates that participants used more administrative functions). The log file was processed using a custom-programmed script (available on request) written in the Perl programming language. A schematic overview of the way the Perl script processed the log file is shown in Figure 2.

### Variable Creation and Factor and Cluster Analysis

As the basis for our typology, we extracted 41 variables derived from the log files that quantify: (1) the number of times patients used individual portal functions during the study period, and (2) the frequency, consistency, duration, and intensity of use (Table 1). We defined frequency on the basis of the total number of sessions during the study period and on the total number of “hit-days”. A hit-day is defined as any day on which a patient accesses the portal, regardless of the number of individual sessions on a given day [26]. Because session counts alone do not characterize use over a longer-term period (eg, a user could have many sessions during a single month and then never use the portal again), we defined a measure of consistency to distinguish users who might have a similar number of sessions overall, but with a different distribution across the study period. Similar to the concept of a hit-day, we measured consistency as the total number of hit-months, which, in turn, were defined as any individual month in which a patient had at least one portal session (eg, 12 hit-months meant that a user accessed the portal at least one time during each month of the study period). Intensity of use was defined as the number of functions accessed by a user during an individual session, as well as by the average page view length (ie, the average number of minutes between the time a user clicks on a link to a specific portal function and the time when they click to go to the next function or to log out of the session) and the total number of functions accessed during the study period. Duration was defined by two variables: the average length of an individual session and the total length of all sessions over the course of the study period.

**Table 1:** Variables extracted from the log file.

Variable (Category/Name)		Description/Definition
**Frequency**		
	sess	Total number of sessions during study period
	hitdays	Hit days (days during study period with ≥1 session)
**Consistency**		
	hitmo	Hit months (months during study period with ≥1 session)
**Duration**		
	avg_sess_len_mins	Average session length (minutes)
	tot_len_mins	Total length of all sessions (minutes)
**Intensity**		
	avg_view_mins	Average length of each page view (minutes)
	totfxn	Total number of functions used
	avgfxnses	Average number of functions accessed per session
Administrative use ratio		
	adminfxn	Total administrative functions accessed during study period
	carefxn	Total of care-related functions accessed during study period
	ratioac	Ratio of number of administrative-to-care functions accessed
**Use of individual portal functions (measured as total times accessed during the study period)**		
	labresults	Review specific lab results
	labtests	Review lab tests
	resultcomponentgraphing	Graph specific test results
	encounterreview	Review list of all previous physician visits
	encounterdetails	Review details of specific physician visit
	allergies	Review list of allergies
	immunizations	Review immunization history
	problemlist	Review problem list
	messaging	Review message inbox
	flowsheetreportslist	View list of trackable clinical measures
	flowsheetreportdetails	Graph specific clinical measures (weight, blood pressure)
	healthmaintenance	Review all preventive care reminders
	healthmaintenanceschedule	Review preventive care reminder
	healthsnapshot	Review summary of preventive health information
	histories	Review patient history
	letters	Review list of referral letters
	medication	Review list of current medications
	medicationrenewalrequest	Renew medication via secure message
	patientnotes	Enter notes viewable only by patient
	personlpreferences	Review/update demographic information
	demographics	Update patient demographic info (email address)
	providerdetails	View detailed information about a provider
	addresschangerequest	Update address information
	referralreview	View referrals to other providers
	referralrequest	Request referral
	appt_final	Appointment-related functions (schedule, review, cancel)
	proxyaccessview	View another individual’s medical record
	customerservicerequest	Send message to customer service
	drivingdirections	View directions to physician/specialist office
	switchcontext	Switch to proxy view

We explored typologies in two steps. First, we used principal components factor analysis with a varimax rotation to reduce the 41 variables in the analytic dataset to 10 composite factor scores (results available on request). Second, we conducted a cluster analysis of individual patient factor scores to identify similar types of MyGeisinger users. Cluster analysis encompasses a variety of mathematical methods for classifying groups of similar entities (eg, portal users), often for the development of typologies [27]. We sought to determine whether there are distinct groups of portal users, where similarity within a group is measured by both the number of specific portal functions they use over time and by measures of the frequency, consistency, duration, and intensity of their use. We used a hierarchical agglomerative clustering algorithm that initially places each patient in a separate cluster and then iteratively joins the two most similar clusters. “Similarity” was assessed using Ward’s minimum variance method. The final cluster analysis solution places each patient into one of a set of mutually exclusive groups or “clusters” designed to minimize the differences between patients within a cluster and maximize the differences between patients in all other clusters. Because the cluster analysis is based on variables that describe study participant’s use of the portal over the 12-month course of the study and not on patient-level variables such as age, sex, or health status, the resulting clusters will be based on similarity of portal use patterns, *not* on similarities between patient-specific variables such as age, sex, or health status. Our final typology was developed by summarizing the patient-level data (eg, age, sex, clinical characteristics) and portal use data for distinct groups of portal users identified by the clustering algorithm in order to develop summary descriptions of each group.

Our analysis used an empirical, hierarchical approach [27,28] rather than an iterative partitioning [29] approach because we did not make a priori assumptions about the number of clusters we expected to identify in our dataset. The cubic clustering criterion and pseudo t-statistics were used to make the final determination of the optimal number of user types (ie, clusters) underlying our typology [30]. To minimize the influence of outliers, we calculated the distribution of the total number of sessions for all portal users and removed those individuals (n=24) whose total number of sessions was greater than the 99^th^ percentile of total number of portal session. Factor and cluster analyses were completed using SAS 9.1; all other statistical analyses used Stata 10.1.

## Results

We identified a total of 3297 study participants who met inclusion criteria and were registered MyGeisinger users (“portal registrants”). Of these, 2282 (69.21%) actually logged in and used the portal at least two times (“registered active users”) during the 12-month study period (Table 2). After excluding 24 patients whose total number of sessions was greater than the 99^th^ percentile, 2258 patients were included in the cluster analysis. Of the remaining 1015 registered patients who were classified as “registered non-users”, 183 used the portal for a single session. “Active users” (ie, ≥2 sessions) were more likely to be male. Age distributions, although statistically different, were largely similar between active users, non-users, and non-registered matched controls (Table 2).

**Table 2:** Characteristics of Web portal registrants who access the site at least 2 times compared with non-registrants and registrants who used the site minimally.

Characteristics		Portal registrants (N=1015): Non-users (≤1 session)		Portal registrants (N=2282): Active users (≥2 sessions)		Portal non-registrants (N=1649): Matched controls	
		n	%	n	%	n	%
**Sex**							
	Female	459	45.22	974	42.68	717	43.48
	Male	556	54.78	1308	57.32	932	56.52
**Age** ^a^							
	<44	103	10.15	237	10.39	190	11.52
	45-54	202	19.90	472	20.68	318	19.28
	55-64	295	29.06	755	33.09	488	29.59
	65-74	221	21.77	525	23.01	389	23.59
	75-84	149	14.68	249	10.91	215	13.04
	85+	45	4.43	44	1.93	49	2.97
**Chronic disease**							
	Diabetes only	433	42.66	1071	46.93	748	45.36
	Cardiovascular only	311	30.64	637	27.91	470	28.50
	Heart failure only	48	4.73	66	2.89	63	3.82
	≥2 chronic conditions	223	21.97	508	22.26	368	22.32
Mean Body Mass Index		31.36		31.34		32.4	

^a^ *P*<.01.

Principal components analysis identified 10 factors. Each patient’s factor scores, which represent estimates of the scores study participants would have received on each of the extracted factors if the factors were measured directly, were used in the cluster analysis model [31]. Using the pseudo t^2^ criteria as a guide, we selected an eight-cluster solution. Two major categories of usage measures (Table 3) were used to characterize portal activity for each of the eight clusters over the entire 12-month study period: (1) “portal use” measures (eg, frequency, consistency, duration, and intensity) that characterize overall use during the entire study period, and (2) “functional use” measures that describe the average number of times that members of a cluster used a specific function (eg, electronic messaging, viewing lab results) over the course of the 12-month study period. Each of the eight clusters was distinguished primarily by the constellation of portal use and functional use measures for which the cluster had either the highest or lowest average value relative to every other cluster (Table 3). For example, the largest cluster, number 1, accounted for 41.98% (948/2258) of the population, had the lowest average measure of intensity of use (7.4 functions per session), and had the lowest average use of the majority of individual portal functions (eg, members of this group accessed the lab results function an average of 20.5 times during the study period). In contrast, Cluster 7 members used the proxy access function 13 times more often (on average) than the members of Cluster 5, which had the second highest average proxy use (54.2 vs 4.2 times) during the study period. Cluster 5 had the highest frequency and consistency of use and the highest average use of the function that allowed users to view and track their lab results (Table 3). Table 4 profiles each cluster on the basis of demographic and clinical characteristics.

Based on the usage patterns and the demographic and clinical characteristics of this cohort of patients with chronic conditions, we offer a typology of eHealth users (Table 5). Type 1 members (“eDabblers”) are low frequency and low intensity users. Members of type 2 (“infrequent intense users”) are similar to Type 1 but have the highest intensity of use as measured by the average number of functions that members of this group access each time they use MyGeisinger. Members of Type 3 (“electronic messengers”) are very high users of secure messaging, including requests for referrals and to renew medications.

Type 4 (“appointment preparers”) is distinguished by frequent use of the portal for appointment scheduling, reviewing information on specific doctors, and viewing directions to a specific clinic location, functions that a patient is expected to use prior to an office visit. Type 5 (“lab trackers”) is characterized by its high use of laboratory test review and tracking functions. Type 6 (“biometric monitors”) is distinguished by its use of the function for tracking weight and blood pressure. Type 7 (“proxy moms”) is predominantly female (80%, 12/15), has the youngest average age (39 years), and demonstrates very high use of the proxy function. Type 8 members (“record updaters”) used the email and address update functions.

**Table 3:** Clustering of patients into eight user types based on cluster analysis of Web portal use patterns (total users N=2258).

			Cluster #							
			1	2	3	4	5	6	7	8
**Cluster size**										
	Number in cluster, n		948	561	209	135	94	238	15	58
	Percent in cluster, %		41.98	24.84	9.26	5.98	4.16	10.54	0.66	2.57
**Web portal use measures**										
	**Frequency**									
		Mean number of sessions	18.5	7.8^a^	46.2	46.0	58.2^b^	20.9	53.3	28.8
	**Consistency**									
		Mean hit-days^c^	15.1	6.9^a^	35.2	36.4	43.3^b^	16.7	37.7	22.8
		Mean hit-months^c^	6.4	4.1^a^	9.3	9.3	10.2^b^	6.8	10.2	7.5
	**Duration**									
		Mean session length, minutes	4.7^a^	10.6^b^	6.9	5.2	6.5	7.5	7.1	6.8
		Mean page view length, minutes	0.6	0.7	0.6	0.8	0.6	0.6	1.4^b^	0.5^a^
	**Intensity**									
		Mean number of functions/session	7.4^a^	18.3^b^	10.1	8.3	14.5	14.3	9.9	12.6
	**Administrative vs Care-related use**									
		Mean ratio of administrative:care use	0.3	0.2^a^	0.3	1.3^b^	0.2^a^	0.2	0.7	0.3
		Review specific lab results	13.9^a^	19.2	30.6	32.7	98.0^b^	29.1	31.5	26.2
		Review list of available lab tests	20.5^a^	23.9	43.8	48.3	143.4^b^	38.6	45.5	36.7
		Graph specific lab test results	2.1^a^	3.3	6.1	5.1	17.5^b^	6.0	7.7	5.0
		Review list of all prior provider visits	4.1^a^	6.7	15.5	23.8	29.8^b^	14.3	29.7	12.2
		Review details of prior provider visit	2.7^a^	4.4	10.6	15.6	19.7^b^	9.7	18.9	8.2
		Review electronic message inbox	28.7	14.4^a^	104.7^b^	55.6	68.1	32.8	54.3	43.6
		View list of graphable values (weight, bp)	0.3^a^	0.4	1.2	1.1	1.5	3.1^b^	2.1	0.9
		View specific graphs (weight, bp)	0.4^a^	0.5	1.6	1.7	2.2	4.7^b^	2.7	1.4
		Review past medical history	0.7^a^	1.1	2.4	1.8	5.2^b^	2.5	1.7	2.2
		View received letters	1.4^a^	1.5	6.9^b^	3.0	5.7	2.7	1.7	3.2
		Review list of current meds	6.3^a^	6.7	20.6	16.5	38.4^b^	12.3	17.0	16.6
		Renew med(s) via electronic message	1.9	0.5^a^	5.0^b^	1.9	2.3	1.3	3.3	2.1
		Update email address^e^	0.5	0.3^a^	1.0^b^	0.5	0.7	0.5	0.7	0.8
		Update address^e^	0.1^a^	0.1^a^	0.3	0.2	0.3	0.3	0.5	2.4^b^
		View detailed provider information^e^	0.7^a^	1.1	2.8	10.0^b^	4.5	2.0	1.9	3.5
		Review approved referrals^e^	1.1^a^	1.6	12.5^b^	7.6	12.3	4.3	3.1	5.9
		Request specialty referral^e^	0.3	0.2^a^	5.5 ^b^	2.6	1.9	0.6	1.4	1.3
		Proxy use (view another’s record)^e^	1.3^a^	1.6	4.1	3.1	4.2	2.5	54.2^b^	3.4
		Send message to customer service^e^	0.7	0.4^a^	5.5^b^	1.7	2.5	1.3	1.7	1.6
		View directions to provider’s office^e^	0.2^a^	0.2	0.8	2.9^b^	1.1	0.4	0.5	0.8
		Schedule/change/cancel appointment^e^	16.5	8.8^a^	46.4	93.3^b^	59.2	24.5	41.1	35.3

^a^Lowest value relative to other clusters.

^b^Highest value relative to other clusters.

^c^Number of individual days/months during study period with ≥1 session.

^d^Administrative function (all others classified as care-related).

^e^The average was calculated based on the total number of times a function was used (ie, a portal menu option was clicked) by each patient in the specified cluster and dividing by the number of patients in the cluster. A function could be used multiple times per session. Not all functions accessible via the portal are listed in this Table.

**Table 4:** Characteristics of patients in each of the eight clusters of user types.

		Cluster # (size)							
		1 (n=948)	2 (n=561)	3 (n=209)	4 (n=135)	5 (n=94)	6 (n=238)	7 (n=15)	8 (n=58)
Patient characteristics									
	Mean age, in years	61.0	60.8	61.2	59.3	61.3	57.2	39.5	53.2
	Gender, % female	41.4	39.6	48.3	42.2	44.7	44.5	80.0	48.3
	Mean Body Mass Index	31.1	30.5	31.0	33.0	29.7	35.2	31.8	27.5
Chronic conditions, n (%)									
	Diabetes mellitus	430 (45.4)	254 (45.3)	103 (49.3)	61 (45.2)	44 (46.8)	124 (52.1)	10 (66.7)	32 (55.2)
	Cardiovascular disease	270 (28.5)	175 (31.2)	50 (23.9)	31 (23.0)	27 (28.7)	63 (26.5)	3 (20.0)	13 (22.4)
	Chronic heart failure	25 (2.6)	18 (3.2)	5 (2.4)	4 (3.0)	2 (2.1)	8 (3.4)	1 (6.7)	2 (3.4)
	≥2 Chronic conditions	223 (23.5)	114 (20.3)	51 (24.4)	39 (28.9)	21 (22.3)	43 (18.1)	1 (6.7)	11 (19.0)

**Table 5:** Eight eHealth patient types based on Web portal use patterns.

Cluster/type # (population %)	Label	Key attributes
1 (42%)	eDabblers	Largest cluster
		Shortest average session length
		Second-lowest average number of sessions
		Lowest intensity use
2 (25%)	Infrequent, intense users	Infrequent but meaningful visits (ie, highest intensity of use)
		Lowest frequency (hit days) of use
		Lowest consistency (hit months) of use
		Highest percentage of male users
3 (9%)	Electronic messenger	Highest use of the secure messaging function
		Highest use of the referral review and request functions
		Highest use of the medication renewal function
		Second-lowest average patient activation score
4 (6%)	Appointment preparers	Highest use of appointment scheduling functions
		Highest ratio of administrative-to-care use (only cluster >1.0)
		Highest use of function that displays provider information
		Highest use of function that provides driving directions to clinic
5 (4%)	Lab trackers	Highest use of the lab results and lab test review functions
		Highest frequency and consistency of use
		Lowest administrative-to-care ratio (ie, more care-related use)
		Second-highest average patient activation score, highest average age
6 (11%)	Biometric monitors	Highest use of weight/blood pressure tracking and graphing
		Second-highest average session length
		Lowest use of most portal functions
		Highest average BMI
7 (1%)	Proxy moms	Highest use of proxy functions (ie, view another person’s record)
		Second-highest frequency and consistency of use
		Second-highest use of function to review list/details of office visits
		Highest proportion of female user, lowest average age
8 (3%)	Record updaters	Highest use of email/address updating functions
		Third-lowest frequency and consistency of use
		Shortest average page view time
		Highest average patient activation score

## Discussion

### Principal Findings

The conceptual model for understanding users of eHealth technologies such as portals, and for understanding the link between portal use and changes in patient outcomes, is not adequately developed and is often categorized along a single dimension. The amount of use (eg, number of logins, page views, time online) is frequently evaluated as the dominant mediator of outcomes associated with eHealth interventions [32]. Our data indicate that portal users are highly heterogeneous. Amount of use captures one of a number of dimensions of effective or meaningful use. User phenotypes may capture unique combinations of known and latent reasons for how eHealth is used because patients appear to exhibit distinct patterns of use. These patterns of use (reflected in the groups identified in Table 5) are characterized not solely by “high” or “low” use, but by variability in the frequency, consistency, and intensity of use over time, as well as by the specific features or functions that they tend to use repeatedly over time. By identifying distinct usage patterns, our typology may offer a tool for articulating more robust hypotheses about why patients use eHealth tools (eg, portals, PHRs) and, therefore, the types of outcomes that may be relevant. For example, there is a conceptual rationale for examining the relationship between portal use and clinical outcomes (eg, HbA1c) for “lab trackers”. Patients who monitor their HbA1c may be more likely to reach their clinical goal. However, a similar rationale may not be valid for “appointment preparers” because there is not a clear rationale for expecting that the way they use the portal (to prepare for an appointment) is likely to directly influence a clinical outcome such as HbA1c. We note that the groups identified in Table 5 are characterized by the portal features they tend to use (or not use) over time, but use of functions within an identified group is not exclusive (eg, patients in the “lab tracker” group are also likely to use the secure messaging function even if their overall pattern of use is different from the “secure messengers”). As portals become more prevalent, payers and providers will be concerned about the value provided by these technologies. Value can be defined based on improvements in patient outcomes, patient satisfaction, market share, or as a combination of measures such as return-on-investment. To establish the relationship between value-focused outcomes and portal use, we need to first understand and design measures that account for, or are the result of, the different patterns of use we have identified. Our results should also inform the development of patient-specific measures of meaningful use [33].

Our results indicate that there appear to be naturally occurring groups of portal users in a primary care patient population. We expected that frequency and intensity of portal use could serve as factors that discriminate various types of eHealth users, and this is partially supported by the data. In addition, several other distinguishing features of users are apparent; for example, proxy users represent a distinct group, as do users who focus on administrative versus care-related functions. Our findings are limited by both our patient selection criteria and by the current structure and features of the institution’s portal. However, our results offer a potential guide to areas where portal redesign can foster greater patient engagement and use. Moreover, our data indicate that the “if you build it, they will come” assumption so often associated with HIT may be a false hope, at least for the types of patients studied. Notably, approximately one-third of patients registered to use the portal never actually accessed it during the course of the study period. Even among “active users”, whom we defined as having at least 2 portal sessions during the study period, more than 65% were relatively infrequent and inconsistent in their use of the portal. Polls have consistently found that patients want the ability to use online tools to schedule appointments, communicate with their physician, receive their lab results, and have access to an EHR [3,34]. More than 50% of respondents in one poll said the ability to engage in such online activities would affect their choice of a physician [2]. While the demand appears to exist for Internet-based tools such as a portal, the form and types of interactions allowed by the current generation of tools may not yet be well defined or developed. Moreover, relatively few patients have access to these tools, and even among those who do have access, our data suggest that there remains an opportunity to develop features that foster more substantial engagement.

Our typology offers insight into potential enhancements to better engage, support, and guide patients in health-related activities. We next consider the distinguishing usage features and patterns of each type of eHealth user and identify the enhanced functions and features that are relevant to each group’s specific usage patterns.

The “appointment preparers” present an opportunity to engage these patients in potentially beneficial activities prior to their visit. For example, these users can, via the portal, be invited to complete electronic versions of data collection instruments (eg, administrative forms, patient-reported outcomes) that, if collected at all, are usually administered by paper during the office visit. Engaging patients prior to the visit has the potential to reduce costs by streamlining clinic workflows and to improve quality as additional data relevant to patient care are made available to the physician at the time of the office visit [35]. Similarly, “lab trackers” have a pattern that presents a low-cost, efficient opportunity to improve quality of care by engaging patients in self-management behaviors at a time when the patient has, by virtue of their decision to access their lab data, indicated an interest in their own health.

“Proxy moms” have the highest proportion of individuals with diabetes. Given their relatively young age, it is likely that these users have a dual role, managing their own chronic condition, and as indicated by their use of the proxy function, the care of a child or elderly parent. These users appear to be motivated to use the portal by their role as a caregiver and additional features relevant to this role may enhance engagement and offer a means for more virtual encounters, including joint virtual encounters where both the patient and the caregiver can participate from separate locations.

The secure messaging function was used by patients in all clusters. However, the “electronic messenger” cluster, characterized by the highest use of this function, was relatively small (9.26%, 209/2258). This was surprising given survey data showing strong interest in this feature. Evidence is mixed on portal-based and/or a standalone (ie, without access to medical record data) secure messaging tools, with one randomized controlled study [36] finding no reduction in telephone calls, versus another study finding a reduction in office visits but not in the number of telephone calls to the clinic [37]. Non-randomized studies evaluating the relationship between portal use (including secure messaging) and measures of utilization have shown a range of results, including a reduction in telephone calls [38], an increased use of clinical services [39], an absence of any significant change in face-to-face visits [40], increases in utilization of specialty and emergency department visits among diabetic patients [41], and increases in in-person and telephone clinical services [42]. Our data suggest that the lack of a clear relationship between portal use and calls/visit is not surprising because the messaging function is heavily used by only a small subset of patients. Earlier studies may fail to show an effect because the messaging function is either not targeted to appropriate user types, the targeted user base is too small to show an effect, or the function is not designed with other features that can increase interest in the use of virtual rather than in-person encounters.

In this study, we chose patients as the unit of analysis. The clustering algorithm identifies groups of similar patients based largely on the “bundle” of different portal functions they use over the course of the study period. Individual patients in one typological group, however, are likely to engage in behaviors associated with other typological groups (eg, lab trackers may also use secure messaging). An alternative approach that should be considered for future research is to consider “sessions” as the unit of analysis. In this case, the clustering algorithm will identify whether there are distinct types of sessions (as opposed to patients) characterized by the use of certain portal functions alone or in combination (eg, secure messages and laboratory results review), and patients can be described on the basis of the types of sessions that they use over time, which may be associated with the need for clinical services, disease severity, demographic characteristics, and other factors.

Although beyond the scope of this study, it is possible to determine which patient characteristics predict a patient’s eHealth user type. Such predictive capabilities will allow organizations to develop targeted approaches to engaging different segments of their population with messages and incentives that can motivate eHealth adoption and use. It may also spur the development of new types of technologies. Many of the currently installed portals function primarily as a read-only view of the data in an individual’s medical record. Although we have described the potential to improve outcomes through a better understanding of the way patients use portals, many of the advances we outlined (eg, using the portal to collect pre-visit data from “appointment preparers”) require functionality not available in the current generation of deployed portals.

### Limitations

This study is subject to several limitations. We have speculated about the relationship between portal use, cluster types, and outcomes; however, conducting a detailed assessment of outcomes and the relationship to our typology was beyond the scope of this study. Data from this study were collected from 2005-2006. Although Geisinger’s portal has changed relatively little in terms of the overall core functionality offered to patients (eg, secure messaging, laboratory results), we believe that over the past 7 years patients have likely become more familiar and comfortable with eHealth tools like the portal. It is likely that this familiarity would, if we re-ran the analysis using data from 2012-2013, change the frequency and consistency with which patients use the portal. Because our typology is based both on the features used and how they are used over time, it is possible that Cluster 1 (“eDabblers”), which is defined by relatively low use, would be smaller, although it is hard to know if/how these users would be distributed among the other clusters. Although the data are older, Geisinger was an early adopter of the patient portal and we believe that the results are relevant to the many health care systems that are implementing EHRs and portals in response to meaningful use incentives.

Our analysis focused on use of MyGeisinger, and our data sources did not include other measures of non-portal patient activity such as office visits, telephone calls, or hospital admissions. This limitation precludes the ability to explore the relationship between portal use and “real-world” office or telephone utilization. We also focused only on patients with chronic disease because we expected that they would have reasonable cause to use the portal repeatedly over time. Our typology cannot be reliably extrapolated to patients without chronic disease because the motivation to use the portal and utility of specific functions is likely to be different from chronically ill patients.

In our study, there are unmeasured provider behaviors (eg, quality and timeliness of provider and staff responses to secure messages), clinic-level behaviors (eg, scheduling and phone practices), and system-wide activities (eg, broadcast and/or targeted preventive care reminders sent to patients) that may have impacted whether and how often patients use the portal. In subsequent analyses, it will be important to incorporate measures of these behaviors and assess their impact on the size, number, and nature of user types identified by our method. Although the portal functions we analyzed are typical of many portals, our typology will need to be updated as current generation portals evolve to provide new and/or more advanced functions. We were limited in our ability to fully characterize cluster members using demographic and EHR data. Notably, like Roblin et al, we did not find evidence of an age disparity in terms of portal use by older patients; more than one-third of portal users (Table 2) were 65 years or older [43]. Some of the naturally occurring variability in portal use may be due to differences in disease severity or physician practice, and these factors should be explored in subsequent studies. To validate our findings, we used a method similar to Coste et al in which we re-ran the analysis on 10 random subsamples of the entire population [44]. We also re-ran the analysis using a partitioning cluster algorithm (k-means), which should replicate the results of the hierarchical approach if the hierarchical approach accurately identified the structure of the underlying data. Both validation approaches yielded acceptable results. However, we consider our results to be a preliminary typology that will likely be refined by similar research using different populations and different types of portals. Regardless of whether our typology is replicated in different populations, our results suggest that Web server log files can serve as a valuable secondary data source for eHealth services research.

The method we have described can be applied more broadly to studies of other types of eHealth technologies. For example, the “lifelong personal health record” described by Barbarito et al, as well as other personal health record systems, may have novel usage patterns because data are owned by the patient rather than a specific health care system (as are many of today’s portals) and the potential for a longitudinal, provider-agnostic view may present new use cases from the patient’s perspective [45].

### Conclusion

Our preliminary typology offers a guide to developing additional features and functionalities that can support patients in their meaningful use of online health-related tools. By identifying distinct patterns of use that may be linked to relevant outcomes, our typology can form a framework around which to design future research focused on the next generation of burgeoning eHealth technologies.

## Abbreviations

BMI: body mass index
EHR: electronic health record
GC: Geisinger Clinic
HIT: health information technology
ICD: International Classification of Diseases
PCP: primary care physician
PHR: personal health record
